# Self-assembly of heteroleptic dinuclear metallosupramolecular kites from multivalent ligands via social self-sorting

**DOI:** 10.3762/bjoc.11.79

**Published:** 2015-05-08

**Authors:** Christian Benkhäuser, Arne Lützen

**Affiliations:** 1 Kekulé-Institute of Organic Chemistry and Biochemistry, University of Bonn, Gerhard-Domagk-Str. 1, D-53121 Bonn, Germany

**Keywords:** metal complexes, multivalency, self-assembly, self-sorting, supramolecular chemistry, Tröger's base

## Abstract

A Tröger's base-derived racemic bis(1,10-phenanthroline) ligand (*rac*)-**1** and a bis(2,2'-bipyridine) ligand with a central 1,3-diethynylbenzene unit **2** were synthesized. Each of these ligands acts as a multivalent entity for the binding of two copper(I) ions. Upon coordination to the metal ions these two ligands undergo selective self-assembly into heteroleptic dinuclear metallosupramolecular kites in a high-fidelity social self-sorting manner as evidenced by NMR spectroscopy and mass spectrometry.

## Introduction

**Scheme 1:**
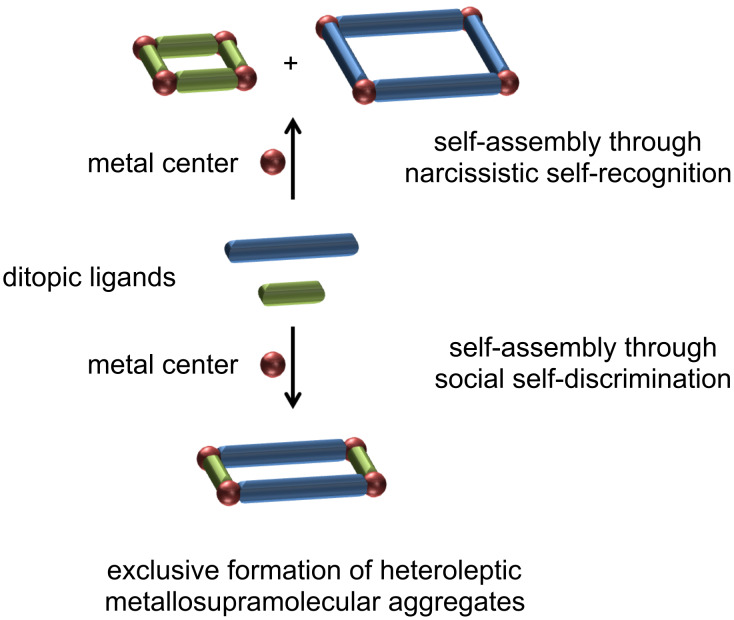
Schematic representation of self-sorting effects in metallosupramolecular self-assembly processes.

Self-assembly of defined aggregates from multicomponent mixtures through self-sorting effects has become an important issue in supramolecular chemistry [1–5]. Such self-sorting can either occur in a social self-discriminating or a narcissistic self-recognition manner (Scheme 1). In general, geometrical size and shape complementarity are used to ensure high-fidelity self-sorting.

This strategy has proven to be very successful for the formation of homoleptic complexes through self-recognition [1–4]. However, self-assembly processes of metallosupramolecular aggregates that integrate more than one type of bridging ligand and/or one type of metal ion into an assembly are even more attractive since they allow access to much more complex supramolecular architectures than homoleptic systems do. Unfortunately, the selective formation of heteroleptic complexes from a mixture of different multivalent ligands bridging two or more metal ions is more challenging and there is only a limited number of reliable protocols available yet [6–7]. These comprise (i) topological control pioneered by J. P. Sauvage [8], (ii) steric control as first established by M. Fujita [9] and P. J. Stang [10] using pyridine and lutidine-based ligands or in M. Schmittel’s HETPHEN [11], HETTAP [12], and HETPYP concepts [13], (iii) metal coordination specifics as pioneered by J.-M. Lehn with metal centers that prefer five-fold coordination [14], or (iv) charge-separation effects as utilized by P. J. Stang [15].

**Scheme 2:**
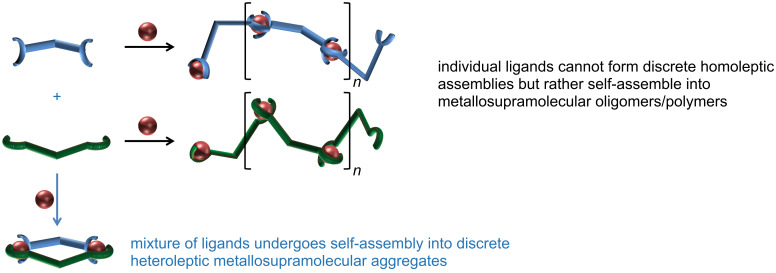
Schematic representation of our approach to discrete heteroleptic oligonuclear metallosupramolecular aggregates in a social self-sorting manner.

As part of our ongoing efforts to develop general guidelines for the (diastereo)selective self-assembly of metallosupramolecular aggregates from multivalent rigid concave ligand structures through (chiral) self-sorting processes [16–23], we were wondering whether we could yet establish another approach to achieve the formation of heteroleptic metallosupramolecular assemblies in a social self-sorting manner as outlined in Scheme 2.

The basic idea is to design multivalent ligands that do not show a (high) tendency to form discrete oligonuclear homoleptic aggregates but rather form metallosupramolecular polymeric structures when mixed with suitable metal ions. In such a scenario the formation of discrete heteroleptic aggregates might become very favorable when such ligands are used in a multicomponent mixture as the formation of discrete macrocyclic or cage-like aggregates is usually entropically more favorable as long as one works in a medium concentration range because the maximum occupancy rule [24] is obeyed which open-chain oligomeric or polymeric species do not do.

## Results and Discussion

### Design and synthesis

**Figure 1:**
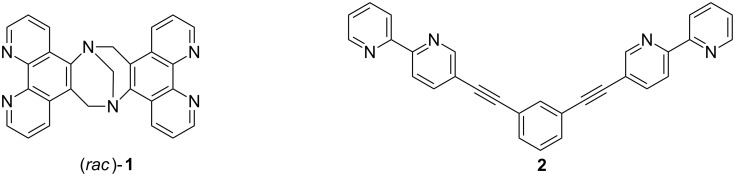
Tröger’s base-derived bis(phenanthroline) ligand (*rac*)-**1** and bis(bipyridine) ligand **2**.

Our strategy asks for the design of rigid multivalent ligands that present their metal binding sites in a way that the formation of discrete macrocyclic or cage-like homoleptic metal complexes is (almost) prevented when they are mixed with a suitable transition metal ion. As the metal ions we chose copper(I) ions which prefer a tetrahedral coordination sphere by two chelating ligands with N-donor centers such as 2,2'-bipyridines or 1,10-phenanthrolines. Connecting two of these ligands with a concave or V-shaped building block with a rather large bent angle should then prevent the formation of discrete oligonuclear cyclic assemblies due to the fact that the chelating units cannot be arranged in the favorable tetrahedral coordination of the copper ions without putting a considerable amount of steric strain into the aggregate. In the search for ligand structures that fulfill these requirements we came up with ligands **1** and **2** that are depicted in Figure 1.

Ligand **1** has a very rigid twisted V-shaped structure that presents its phenanthroline units in a way that is very unfavorable for the formation of discrete metallomacrocyclic assemblies upon coordination to a metal ion that prefers a tetrahedral coordination by two chelating ligands. The same is true for ligand **2** which adopts a flat conformation to maximize π-conjugation. To form a macrocyclic assembly the bipyridine units in this ligand would have to rotate around the alkynyl linkage by about 90° relative to the central *m*-substituted benzene. This is possible, but not favorable, although the barrier for the rotation around the alkynyl linkage is rather low. In addition the ligand would also have to adopt a more strained conformation with considerably bent alkynyl linkages and/or considerably distorted tetrahedral coordination spheres around the metal centers. This makes the two ligands complementary, and hence, prone to the formation of a heteroleptic dinuclear metallosupramolecular assembly with tetrahedral-coordinated metal ions because they are preorganized in a way that they present their metal binding sites in an almost orthogonal fashion and in the right distance.

**Scheme 3:**
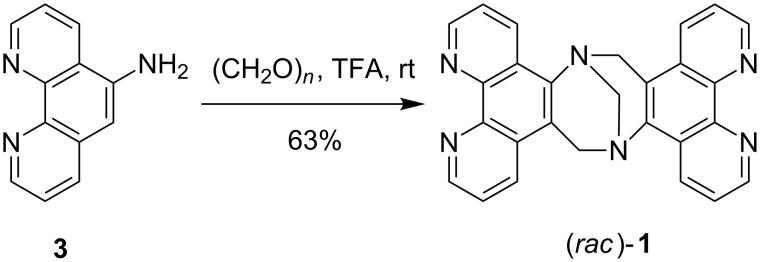
Synthesis of chiral bis(phenanthroline) ligand (*rac*)-**1** from **3**.

In fact, (*rac*)-**1** has been synthesized before by E. Yashima from commercially available 5-aminophenanthroline (**3**) [25]. However, when we employed the reaction conditions that K. Wärnmark [26] has developed for the synthesis of other functionalized Tröger’s base derivatives we were able to increase the yield of (*rac*)-**1** considerably to 63% (Scheme 3).

**Scheme 4:**
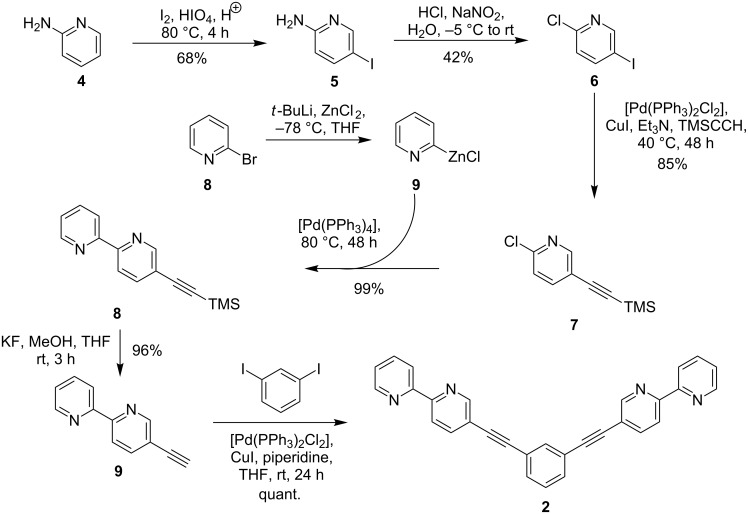
Synthesis of bis(bipyridine) ligand **2** from 2-aminopyridine (**4**).

The synthesis of **2** was achieved in six consecutive steps starting from commercially available 2-aminopyridine (**4**) (Scheme 4) following mostly literature-known protocols. The electrophilic iodination of aminopyridine **4** gave iodide **5** in good yield. Compound **5** was then subjected to a Sandmeyer-like chlorination to **6** which in turn was transformed in a Sonogashira reaction with (trimethylsilyl)acetylene into **7** in a yield of 85% [27]. Alkyne **7** was then subjected to a Negishi reaction with 2-bromopyridine (**8**) derived zinc organyl **9** to give the silyl-protected ethynylated bipyridine **10** in excellent yield of 99% which was subsequently desilylated under standard conditions to give terminal alkyne **11** in 96% yield [28]. Finally, a two-fold Sonogashira reaction with 1,3-diiodobenzene afforded the desired bis(2,2’-bipyridine) ligand **2** in quantitative yield.

### Metal coordination

**Figure 2:**
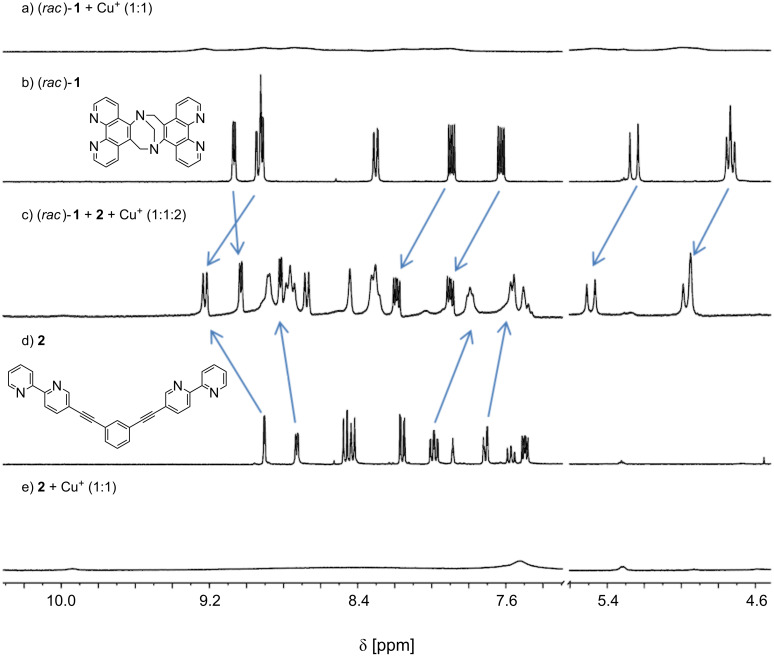
NMR spectra (500.1 MHz in DMSO-*d*_6_ at 295 K) of free ligands b) (*ra*c)-**1** and c) **2**; 1:1 mixtures of ligands a) (*ra*c)-**1** and e) **2** with Cu^+^ salts and c) the resulting NMR of a mixture of these. Arrows indicate the complexation induced shifts of selected signals upon formation of the heteroleptic dinuclear complex.

After the successful synthesis we prepared a DMSO solution of copper(I) ions, added it to the ligands (*rac*)-**1** and **2** each in a 1:1 ratio, and compared the resulting spectra to those of the free ligands (Figure 2b and Figure 2d). In both cases the colors of the solutions turned almost immediately to dark red-brown which indicates the formation of copper(I) complexes. As expected, however, NMR spectroscopic (Figure 2a and Figure 2e) and ESI mass spectrometric studies clearly showed that these complexes are oligomeric or polymeric in nature since no discrete smaller aggregates could be detected.

**Figure 3:**
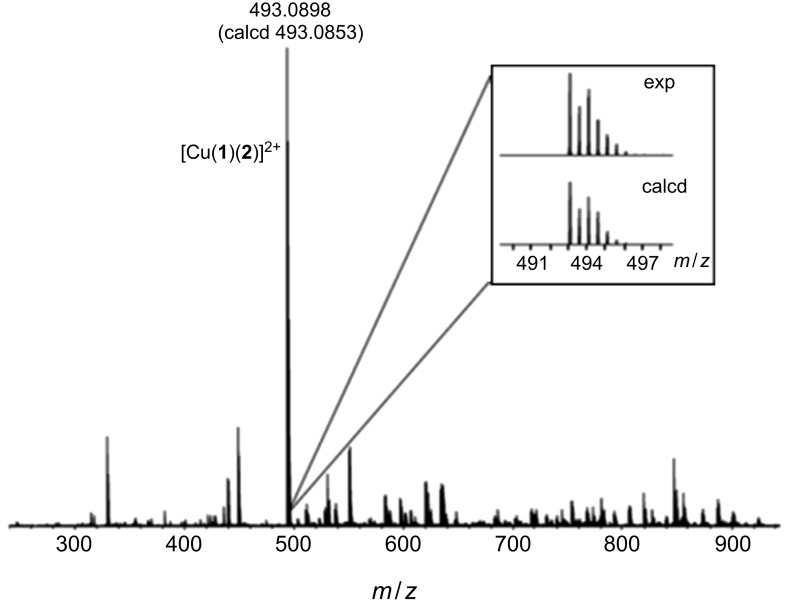
ESI mass spectrum (positive ion mode) of a 1:1:2 mixture of (*rac*)-**1**, **2**, and CuBF_4_ sprayed from a 10^−5^ mM solution in acetone/DMSO 100:1.

We next mixed the two solutions of the non-defined homoleptic complexes (*rac*)-**1** and **2** and observed a set of sharp and considerably shifted signals in the NMR spectrum. This indicated an almost instantaneous rearrangement of the complexes resulting in the self-assembly of a well-defined discrete heteroleptic dinuclear metallosupramolecular assembly with a kite-like structure in a high-fidelity self-sorting manner (Figure 2c). The composition of the assembly was confirmed by ESIMS (Figure 3). Of course, the same result was also obtained when two equivalents of the copper(I) salt in DMSO were added to an equimolar mixture of the ligands (*rac*)-**1** and **2**. It should be noted, that the NMR spectrum still shows some broadened signals (e.g., around 9.8, 7.5, and 5.3 ppm) which might indicate that some minor amounts of oligomers/polymers are still existing. However, the intensity of these signals was so low, that we could not assign a diffusion coefficient to them in a 2D-DOSY experiment to corroborate this assumption.

**Scheme 5:**
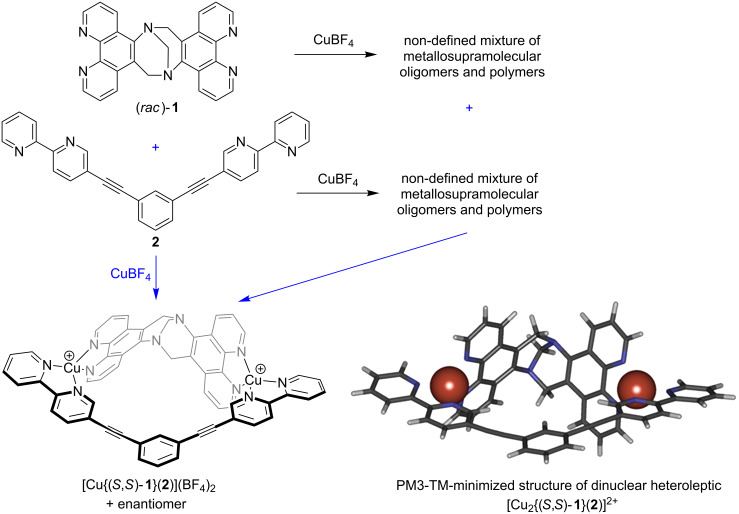
Summary of the coordination behavior of the two ligands **1** and **2** and their equimolar mixture towards copper(I) ions.

Unfortunately, we were not able to grow suitable single crystals of this complex that could be analyzed by X-ray diffraction. Nevertheless the experimental evidence provided by the NMR and MS investigations clearly indicate the formation of the desired heteroleptic complex [Cu_2_(**1**)(**2**)](BF_4_)_2_ in racemic form. Scheme 5 summarizes the coordination behavior of the two ligands **1** and **2** and their mixture towards copper(I) ions.

## Conclusion

In summary, we have synthesized two concave or V-shaped multivalent ligands − a dissymmetric bis(phenanthroline) ligand (*rac*)-**1** based on the Tröger's base scaffold in its racemic form and a bis(2,2'-bipyridine) ligand **2**. Upon coordination to copper(I) ions none of these ligands alone self-assembles into discrete homoleptic oligonuclear metallosupramolecular aggregates. When mixed in an equimolar ratio, however, these ligands undergo highly selective self-assembly into heteroleptic dinuclear metallosupramolecular [Cu_2_(**1**)(**2**)](BF_4_)_2_ kites upon coordination to copper(I) ions in a high-fidelity social self-sorting process. This process is completive according to the classification of M. Schmittel [4] because all of the components of the mixture are used to form the supramolecular aggregates. However, it is not integrative following the classification of self-sorting processes according to C. A. Schalley [5,29–30] because not all of the components present in the mixture form a single type of supramolecular aggregate but they rather form a racemic mixture of chiral aggregates in our case. Hence, the whole process occurs in a social, non-integrative, 2^4,4^-fold (3) completive self-discriminating manner according to M. Schmittel’s classification [4]. This represents a promising strategy for the rational synthesis of heteroleptic metallosupramolecular aggregates from multivalent ligands that we will explore further in the future.

## Experimental

Reactions under inert gas atmosphere were performed under argon using standard Schlenk techniques and oven-dried glassware prior to use. Thin-layer chromatography was performed on aluminum pre-coated TLC plates (silica gel 60F_254_) from Merck. Detection was carried out under UV light (254 and 366 nm). Products were purified by column chromatography on silica gel 60 (70–230 mesh) from Merck. The ^1^H and ^13^C NMR spectra were recorded on a Bruker Avance 500 spectrometer at 298 K, at 500.1 and 125.8 MHz, or a Bruker AM 400 at 293 K, at 400.1 MHz and 100.6 MHz, respectively. ^1^H NMR and ^13^C NMR chemical shifts of the ligands **1** and **2** are reported on the δ scale (ppm) relative to residual non-deuterated solvent (^1^H) or relative to deuterated solvent (^13^C), respectively, as the internal standard. Signals were assigned on the basis of ^1^H, ^13^C, HMQC, and HMBC NMR experiments. For the numbering of the individual nuclei please see the numbering in the structural formula given for the individual compounds. Unfortunately, we were not able to obtain a sufficiently resolved ^13^C NMR spectrum of the heteroleptic complex. Mass spectra were recorded with a microOTOF-Q or an Apex IV FT-ICR spectrometer from Bruker. Elemental analyses were carried out with a Heraeus Vario EL. Most solvents were dried, distilled, and stored under argon according to standard procedures. 2-Amino-5-iodopyridine (**5**) [27], 2-chloro-5-iodopyridine (**6**) [27], 2-chloro-5-{(trimethylsilyl)ethynyl}pyridine (**7**) [27], 5-{(trimethylsilyl)ethynyl}-2,2'-bipyridine (**10**) [28], and 5-ethynyl-2,2'-bipyridine (**11**) [28] were prepared according to literature known procedures.

**(*rac*)-6*H*,16*H*-5,15-Methanodi-1*N*,10*N*,11*N*,20*N*-phenanthro[5’,6’-*b*,5’’,6’’-*f*][1,5]diazocine ((*rac*)-1):** 5-Aminophenanthroline (**3**, 1 g, 5.1 mmol) and paraformaldehyde (323 mg, 10.8 mmol, 2.1 equiv) were mixed in a round-bottomed flask in the dark and cooled with an ice bath. Trifluoroacetic acid (15.2 mL, 133.2 mmol, 26 equiv) was added and the resulting mixture was stirred for 18 h in the dark. After that time the reaction mixture was added drop wise into water (200 mL). After cooling to room temperature the resulting suspension was carefully neutralized with a 6 N aq NaOH solution. The precipitate was collected by filtration, and dried in vacuum. The product was recrystallized from acetone to give the solid product (967 mg, 63%).


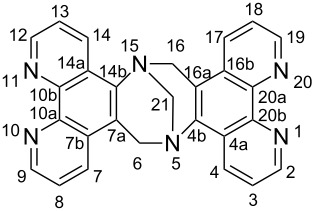


^1^H NMR (400 MHz, DMSO-*d*_6_) δ 9.07 (dd, *J*_8,9_ and *J*_18,19_ = 4.3 Hz, *J*_7,9_ and *J*_17,19_ = 1.7 Hz, 2H, H-9, H-19), 8.94 (dd, *J*_7,8_ and *J*_17,18_ = 8.3 Hz, *J*_7,9_ and *J*_17,19_ = 1.7 Hz, 2H, H-7, H-17), 8.92 (dd, *J*_2,3_ and *J*_12,13_ = 4.5 Hz, *J*_2,4_ and *J*_12,14_ = 1.6 Hz, 2H, H-2, H-12), 8.30 (dd, *J*_3,4_ and *J*_13,14_ = 8.5 Hz, *J*_2,4_ and *J*_12,14_ = 1.6 Hz, 2H, H-4, H-14), 7.89 (dd, *J*_7,8_ and *J*_17,18_ = 8.3 Hz, *J*_8,9_ and *J*_18,19_ = 4.3 Hz, 2H, H-8, H-18), 7.63 (dd, *J*_2,3_ and *J*_12,13_ = 4.5 Hz, *J*_3,4_ and *J*_13,14_ = 8.5 Hz, 2H, H-3, H-13), 5.26 (d, *J*_6exo,6endo_ and *J*_16exo,16endo_ = −17.6 Hz, 2H, H-6_exo_, H-16_exo_), 4.74 (s, 2H, H-21), 4.73 (d, *J*_6exo,6endo_ and *J*_16exo,16endo_ = −17.6 Hz, 2H, H-6_endo_, H-16_endo_) ppm; ^1^H NMR (400 MHz, CDCl_3_) δ 9.24 (dd, *J*_8,9_ and *J*_18,19_ = 4.4 Hz, *J*_7,9_ and *J*_17,19_ = 1.7 Hz, 2H, H-9, H-19), 9.10 (dd, *J*_2,3_ and *J*_12,13_ = 4.4 Hz, *J*_2,4_ and *J*_12,14_ = 1.7 Hz, 2H, H-2, H-12), 8.94 (dd, *J*_7,8_ and *J*_17,18_ = 8.3 Hz, *J*_7,9_ and *J*_17,19_ = 1.7 Hz, 2H, H-7, H-17), 8.09 (dd, *J*_3,4_ and *J*_13,14_ = 8.5 Hz, *J*_2,4_ and *J*_12,14_ = 1.7 Hz, 2H, H-4, H-14), 7.83 (dd, *J*_7,8_ and *J*_17,18_ = 8.3 Hz, *J*_8,9_ and *J*_18,19_ = 4.3 Hz, 2H, H-8, H-18), 7.58 (dd, *J*_2,3_ and *J*_12,13_ = 4.4 Hz, *J*_3,4_ and *J*_13,14_ = 8.5 Hz, 2H, H-3, H-13), 5.24 (d, *J*_6exo,6endo_ and *J*_16exo,16endo_ = −16.9 Hz, 2H, H-6_exo_, H-16_exo_), 4.78 (s, 2H, H-21), 4.73 (d, *J*_6exo,6endo_ and *J*_16exo,16endo_ = −16.9 Hz, 2H, H-6_endo_, H-16_endo_) ppm; ^13^C NMR (100 MHz, CDCl_3_) δ 150.1 (C-9, C-19), 149.1 (C-2, C-12), 146.5, 144.5 (C-10b, C-20b, C-4b, C-14b), 141.3 (C-7, C-17), 131.8 (C-7b, C-16b), 130.1 (C-10a, C-20a), 127.0 (C-7a, C16a), 126.0 (C-3, C-13), 123.6, 123.5 (C-4, C-14, C-8, C-18), 120.0 (C-4a, C-14a), 68.5 (C-21), 53.3 (C-6, C-16); ESIMS (pos.) *m*/*z*: 449.1 [M + Na]^+^. These analytical data are in accordance with the literature data [25].

**1,3-Bis(2,2’-bipyridin-5-ylethynyl)benzene (2):** A two-necked round-bottomed flask was charged with 5-ethynyl-2,2’-bipyridine (**11**, 109 mg, 0.6 mmol, 2 equiv), 1,3-diiodobenzene (100 mg, 0.3 mmol), [Pd(PPh_3_)_2_Cl_2_] (5.32 mg, 2.5 mol %), and copper(I) iodide (1.44 mg, 2.5 mol %) and flushed with argon. Dry THF (15 mL) and dry piperidine (5 mL) were added and the resulting mixture was stirred at room temperature for 24 h. After that time the precipitate was collected and washed three times with THF to afford the desired solid product in sufficient purity (130 mg, quant).


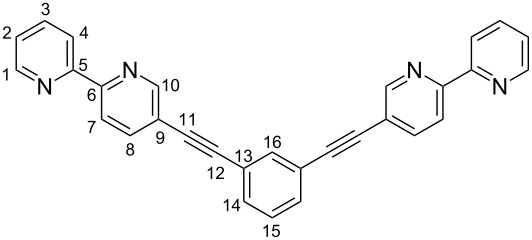


^1^H NMR (400 MHz, CDCl_3_) δ 8.83 (dd, *J*_8,10_ = 2.1 Hz, *J*_7,10_ = 0.9 Hz, 2H, H-10), 8.70 (ddd, *J*_1,2_ = 4.8 Hz, *J*_1,3_ = 1.8 Hz, *J*_1,4_ = 0.9 Hz, 2H, H-1), 8.48–8.40 (m, 4H, H-4, H-7), 7.95 (dd, *J*_7,8_ = 8.3 Hz, *J*_8,10_ = 2.1 Hz, 2H, H-8), 7.84 (ddd, *J*_1,3_ = 1.8 Hz, *J*_2,3_ = 7.6 Hz, *J*_3,4_ = 7.8 Hz, 2H, H-3), 7.80 (dd, *J*_14,16_ = 1.6 Hz, *J*_15,16_ = 0.8 Hz, 1H, H-16), 7.57 (dd, *J*_14,15_ = 7.7 Hz, *J*_14,16_ = 1.6 Hz, 2H, H-14), 7.40 (dd, *J*_14,15_ = 7.7 Hz, *J*_15,16_ = 0.8 Hz, 2H, H-15), 7.33 (ddd, *J*_1,2_ = 4.8 Hz, *J*_2,3_ = 7.6 Hz, *J*_2,4_ = 1.2 Hz, 2H, H-2) ppm; ^13^C NMR (100 MHz, CDCl_3_) δ 155.1 (C-6), 154.3 (C-5), 151.9 (C-10), 148.9 (C-1), 139.7 (C-8), 137.8 (C-3), 135.0 (C-16), 132.1 (C-14), 128.9 (C-15), 124.3 (C-2), 123.3 (C-7), 121.8 (C-4), 120.8 (C-13), 120.4 (C-9), 92.8(C-11), 87.3 (C-12); ESIMS (pos.) *m*/*z*: 457.1 [M + Na]^+^, 435.2 [M + H]^+^; HRMS–ESI (*m*/*z*): [M + Na]^+^ calcd for C_30_H_18_N_4_Na, 457.1424; found, 457.1420; Anal. calcd for C_30_H_18_N_4_·H_2_O: C, 79.36; H, 4.46; N, 12.38; found: C, 79.87; H, 4.95; N, 12.48 (%).

**Preparation and characterization of the metal complexes:** [Cu(H_3_CCN)_4_]BF_4_ (6.3 mg, 20 µmol) were dissolved in DMSO-*d*_6_ (1 mL). This solution (500 µL) were added to (*rac*)-**1** (4.26 mg, 10 µmol) and the remaining 500 µL of the solution were added to **2** (4.34 mg, 10 µmol), respectively. The resulting solutions were characterized by NMR. For the ESIMS studies small aliquots of these solutions (10 µL) were taken and diluted with acetone (1 mL). Subsequently the DMSO solutions were mixed and again characterized by NMR. For the ESIMS study a small aliquot of the mixed solution (10 µL) was taken and diluted with acetone (1 mL).

## References

[1] Gosh S, Isaacs L, Miller B L (2010). Complex self-sorting systems. Dynamic Combinatorial Chemistry.

[2] Osowska K, Miljanić O Š (2011). Synlett.

[3] Safont-Sempere M M, Fernández G, Würthner F (2011). Chem Rev.

[4] Saha M L, Schmittel M (2012). Org Biomol Chem.

[5] He Z, Jiang W, Schalley C A (2015). Chem Soc Rev.

[6] De S, Mahata K, Schmittel M (2010). Chem Soc Rev.

[7] Saha M L, Neogi S, Schmittel M (2014). Dalton Trans.

[8] Dietrich-Buchecker C O, Sauvage J P, Kintzinger J P (1983). Tetrahedron Lett.

[9] Yoshizawa M, Nagao M, Kumazawa K, Fujita M (2005). J Organomet Chem.

[10] Zhao L, Northrop B H, Zheng Y-R, Yang H-B, Lee H J, Lee Y M, Park J Y, Chi K-W, Stang P J (2008). J Org Chem.

[11] Schmittel M, Ganz A (1997). Chem Commun.

[12] Schmittel M, Kalsani V, Kishore R S K, Cölfen H, Bats J W (2005). J Am Chem Soc.

[13] Schmittel M, He B, Fan J, Bats J W, Engeser M, Schlosser M, Deisenroth H-J (2009). Inorg Chem.

[14] Hasenknopf B, Lehn J-M, Baum G, Fenske D (1996). Proc Natl Acad Sci U S A.

[15] Chi K-W, Addicott C, Arif A M, Stang P J (2004). J Am Chem Soc.

[16] Rang A, Nieger M, Engeser M, Lützen A, Schalley C A (2008). Chem Commun.

[17] Weilandt T, Kiehne U, Schnakenburg G, Lützen A (2009). Chem Commun.

[18] Dalla Favera N, Kiehne U, Bunzen J, Hytteballe S, Lützen A, Piguet C (2010). Angew Chem.

[19] Weilandt T, Kiehne U, Bunzen J, Schnakenburg G, Lützen A (2010). Chem – Eur J.

[20] Gütz C, Hovorka R, Schnakenburg G, Lützen A (2013). Chem – Eur J.

[21] Gütz C, Hovorka R, Stobe C, Struch N, Topić F, Schnakenburg G, Rissanen K, Lützen A (2014). Eur J Org Chem.

[22] Hovorka R, Hytteballe S, Piehler T, Meyer-Eppler G, Topić F, Rissanen K, Engeser M, Lützen A (2014). Beilstein J Org Chem.

[23] Meyer-Eppler G, Topić F, Schnakenburg G, Rissanen K, Lützen A (2014). Eur J Inorg Chem.

[24] Krämer R, Lehn J-M, Marquis-Rigault A (1993). Proc Natl Acad Sci U S A.

[25] Yashima E, Akashi M, Miyauchi N (1991). Chem Lett.

[26] Jensen J, Wärnmark K (2001). Synthesis.

[27] Baxter P N W (2000). J Org Chem.

[28] Lützen A, Hapke M (2002). Eur J Org Chem.

[29] Jiang W, Winkler H D F, Schalley C A (2008). J Am Chem Soc.

[30] Jiang W, Schalley C A (2009). Proc Natl Acad Sci U S A.

